# A case of prenatal chronic intestinal pseudo‐obstruction associated with Leigh syndrome

**DOI:** 10.1002/ccr3.1638

**Published:** 2018-06-13

**Authors:** Toshiyuki Itai, Hiroshi Ishikawa, Kenji Kurosawa, Yu Tsuyusaki

**Affiliations:** ^1^ Department of Obstetrics and Gynecology Kanagawa Children's Medical Center Yokohama Kanagawa Japan; ^2^ Department of Genetics Kanagawa Children's Medical Center Yokohama Kanagawa Japan; ^3^ Department of Neurology Kanagawa Children's Medical Center Yokohama Kanagawa Japan

**Keywords:** bowel dilation, chronic intestinal pseudo‐obstruction, Leigh syndrome, prenatal diagnosis

## Abstract

Chronic intestinal pseudo‐obstruction (CIPO) is a major sign of mitochondrial disorders. We present the first reported case of fetal bowel dilation associated with Leigh syndrome. The possibility of CIPO should be taken into consideration even when mild fetal bowel dilation is detected.

## 1 INTRODUCTION

Fetal ultrasound findings of bowel dilation usually suggest mechanical obstruction such as bowel atresia, cystic fibrosis, or meconium peritonitis.^1^ Chronic intestinal pseudo‐obstruction (CIPO) is a rare condition characterized by symptoms of bowel obstruction in the absence of mechanical obstruction.^2^ Compared to CIPO in children and adults, few cases of prenatal CIPO have been reported. We present a case of fetal CIPO associated with Leigh syndrome, which has not been previously reported.

## 2 CASE HISTORY

**Figure 1:**
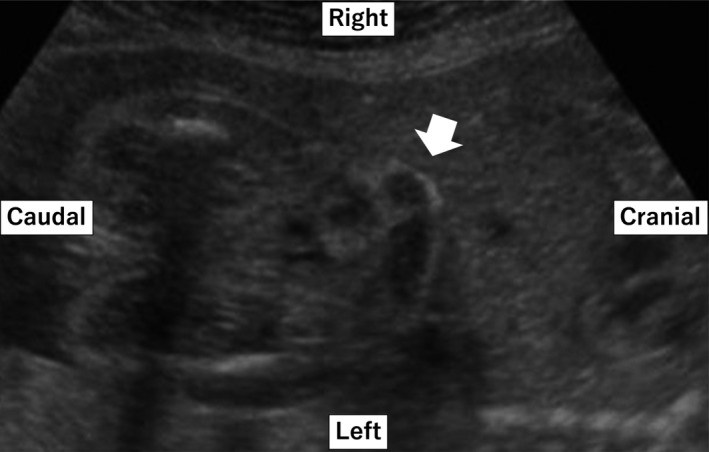
Coronal view of prenatal ultrasound at 24 wk gestation showing hyperechogenic, mildly dilated bowel (arrow)

**Figure 2:**
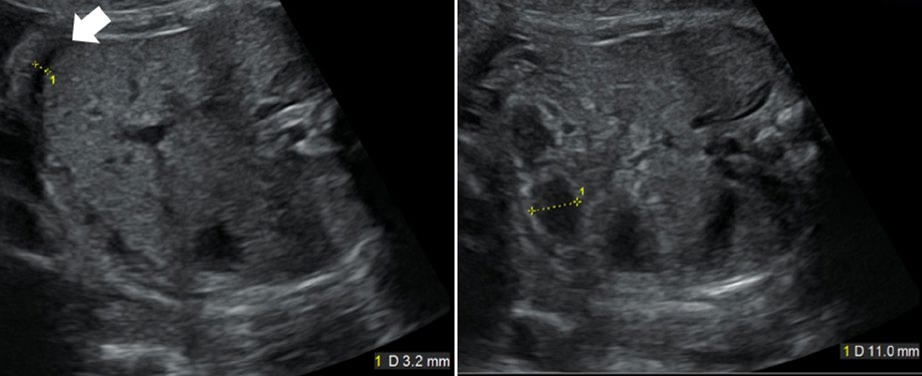
Transverse view of prenatal ultrasound at 31 wk gestation. Right imaging shows mild dilated hyperechogenic transverse colon, and left imaging shows small amount of ascites (arrow)

A 37‐year‐old gravida 2, para 1 (1 spontaneous miscarriage) woman was referred to our hospital at 24 weeks of gestation, who presented with abnormal fetal ultrasound findings of dilated hyperechogenic bowel and a small volume of ascites (Figure 1). Judging from the location of the bowel, we suspected that the dilated bowel was the transverse colon. There was no other organ anomaly. Thus, meconium peritonitis was initially suspected. Dilation of the bowel and ascites worsened slightly until 31 weeks (Figure 2), but subsequently improved; there was no abnormal finding in the fetus’ abdomen at approximately 36 weeks of gestation. At 38 weeks of gestation, the neonate was delivered via normal vaginal delivery. A 2716‐g female neonate was born with APGAR 8/8 and umbilical artery pH 7.33. In the neonatal period, she had no abdominal symptoms but did have some episodes of vomiting, which was not considered abnormal. After the checkup at age 1 month, she stopped visiting our hospital.

**Figure 3:**
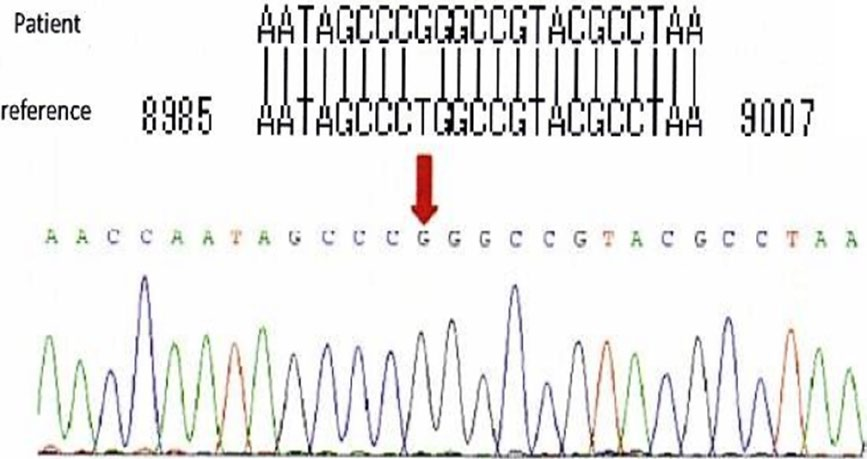
Result of mitochondrial DNA analysis. m8993T>G in the *MT‐ATP6* gene was detected

**Figure 4:**
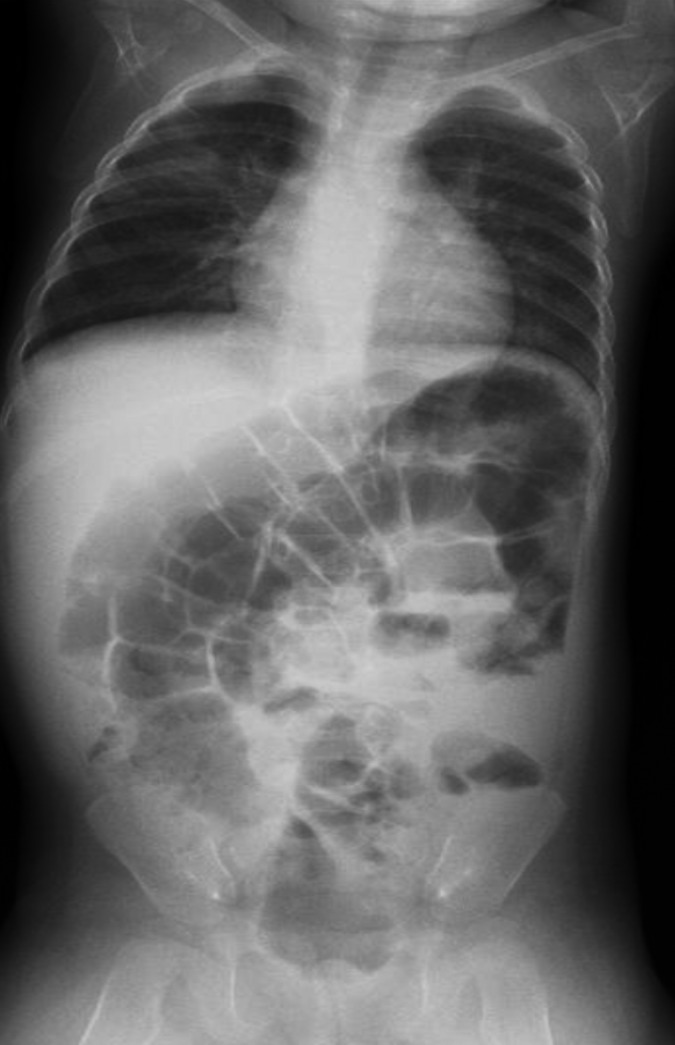
Plain abdominal radiograph at age 12 mo showing enlarged bowel similar to findings of ileus

At 6 months of age, the neonate was brought to our hospital again because of several episodes of vomiting, developmental retardation, and seizure. Elevated serum level of lactate and magnetic resonance imaging findings suggested the diagnosis of Leigh syndrome. After mitochondrial DNA analysis, m8993T>G in the *MT‐ATP6* gene was detected (Figure 3). During follow‐up, she had several episodes of vomiting, and plain abdominal radiography showed dilated bowel similar to that seen in paralytic ileus (Figure 4). From these finding and her episodes of vomiting, we made the diagnosis of CIPO, which is often recognized as a symptom of mitochondrial disease. The infant died of respiratory failure due to brainstem dysfunction at age 14 months. Autopsy was not performed.

## 3 DISCUSSION

Fetal ultrasound findings of bowel dilation usually suggest mechanical obstruction. Nonmechanical bowel obstruction is rare, and only a few cases have been reported, such as those of congenital diarrhea and bowel ischemia.^1^

Chronic intestinal pseudo‐obstruction represents bowel dysmotility in the absence of mechanical obstruction. In Japan, the reported prevalence of CIPO was 3.7 per 1 million children^3^ and 9.0 per 1 million adults.^4^ In the United States, 100 CIPO cases are estimated to be diagnosed annually.^5^ A wide spectrum of disorders can cause CIPO in children and adults, for example, neurological disorders, myopathies, and metabolic diseases, but some cases of CIPO are undetectable.^2^ Despite the fact that more than half of pediatric patients diagnosed with CIPO develop their symptoms in the neonatal period,^3^ very few cases of fetal CIPO were reported.^6^

Compared to that in typical bowel atresia, the degree of dilation in our case seemed inconspicuous. It is suggested that fetal CIPO tends to show slight bowel dilation, possibly due to the absence of oral ingestion during the fetal period. According to Harris et al^7^, the mean and +2 SD colon diameters are 8 mm and 11 mm at 30 weeks and 9 mm and 12 mm at 32 weeks of gestation, respectively. In our case, colon diameter at 31 weeks was 11.0 mm, which was approximately +2 SD above the mean. In addition, we did not refer the case back to the previous hospital because of hyperechogenic bowel wall and small volume of ascites and continued follow‐up of the neonate. There is only a 3‐mm difference between the mean and +2 SD colon diameters at 30 weeks of gestation. Thus, we need to estimate fetal gastrointestinal findings carefully by not only measuring the diameter of the bowel, but also observing other abnormal findings, such as echogenicity of the bowel wall and ascites.

Clinical manifestations of mitochondrial disorders vary widely. Although some affect only a single organ, they can commonly affect multiple organs, including the gastrointestinal tract.^8^, ^9^ The reported abnormal fetal ultrasound findings associated with mitochondrial disorders include periventricular pseudocysts^10^ and skin edema with diminished fetal movements.^11^ Based on these data and our case, if a nonspecific abnormal fetal ultrasound finding is detected, it might be the only feature to suggest a mitochondrial disorder.

Leigh syndrome affects 1 in 40 000 newborns and usually manifests during infancy. Several genetic changes can cause Leigh syndrome; as seen in our case, *MT‐ATP6* gene mutations are the most common mitochondrial DNA mutations in Leigh syndrome.^12^ Similar to other mitochondrial disorders,^8^, ^9^ Leigh syndrome can lead to CIPO, which is usually diagnosed as a first sign of the disease.^12^ Thus, it is conceivable that a fetus with Leigh syndrome would show CIPO, although there has been no case report of fetal CIPO related to Leigh syndrome. This may be because of the low incidence of Leigh syndrome; as shown in our case, it is difficult to suspect CIPO based on findings of slightly dilated and hyperechogenic bowel.

When mild bowel dilation and some additional findings, such as hyperechogenic bowel or ascites, are observed, even if the severity of dilation is mild, CIPO should be considered. In children and adults, fetal chronic intestinal obstruction may occur secondary to various diseases, including mitochondrial disorders, and it should be considered in the prenatal diagnosis of dilated bowel.

## AUTHORSHIP

TI: drafted the manuscript. HI: revised the manuscript. KK: examined mitochondrial DNA analysis of the patient and revised the manuscript. YT: collected the patient's clinical information during her infant period and revised the manuscript. All authors read and approved the final version of the manuscript.

## CONSENT FOR PUBLICATION

Written informed consent was obtained from parents of the patient for publication of this case report.

## CONFLICT OF INTEREST

None declared.

## References

[1] Bulas DI . Bowel abnormalities In: Kline‐Fath BM, Bulas DI, Bahado‐Singh R, eds. Fundamental and Advanced Fetal Imaging. Alphen aan den Rijn, The Netherlands: Wolters Kluwer; 2015:617‐634.

[2] Di Nardo G , Di Lorenzo C , Lauro A , et al. Chronic intestinal pseudo‐obstruction in children and adults: diagnosis and therapeutic options. Neurogastroenterol Motil. 2017;29:e12945. 10.1111/nmo.12945 27683196

[3] Muto M , Matsufuji H , Tomomasa T , et al. Pediatric chronic intestinal pseudo‐obstruction is a rare, serious, and intractable disease: a report of a nationwide survey in Japan. J Pediatr Surg. 2014;49:1799‐1803. 25487487 10.1016/j.jpedsurg.2014.09.025

[4] Iida H , Ohkubo H , Inamori M , Nakajima A , Sato H . Epidemiology and clinical experience of chronic intestinal pseudo‐obstruction in Japan: a nationwide epidemiologic survey. J Epidemiol. 2013;23:288‐294. 23831693 10.2188/jea.JE20120173 PMC3709546

[5] Di Lorenzo C . Pseudo‐obstruction: current approaches. Gastroenterology. 1999;116:980‐987. 10092321 10.1016/s0016-5085(99)70082-x

[6] Shen O , Schimmel MS , Eitan R , Granovsky‐Grisaru S , Rabinowitz RR . Prenatal diagnosis of intestinal pseudo‐obstruction. Ultrasound Obstet Gynecol. 2007;29:229‐231. 17252528 10.1002/uog.3895

[7] Harris RD , Nyberg DA , Mack LA , Weinberger E . Anorectal atresia: prenatal sonographic diagnosis. AJR Am J Roentgenol. 1987;149:395‐400. 3300224 10.2214/ajr.149.2.395

[8] Sekino Y , Inamori M , Yamada E , et al. Characteristics of intestinal pseudo‐obstruction in patients with mitochondrial diseases. World J Gastroenterol. 2012;18:4557‐4562. 22969229 10.3748/wjg.v18.i33.4557 PMC3435781

[9] Chapman TP , Hadley G , Fratter C , et al. Unexplained gastrointestinal symptoms: think mitochondrial disease. Dig Liver Dis. 2014;46:1‐8. 23768727 10.1016/j.dld.2013.04.008

[10] Leshinsky‐Silver E , Lev D , Malinger G , et al. Leigh disease presenting in utero due to a novel missense mutation in the mitochondrial DNA‐ND3. Mol Genet Metab. 2010;100:65‐70. 20202874 10.1016/j.ymgme.2010.02.002

[11] Arnon S , Aviram R , Dolfin T , et al. Mitochondrial DNA depletion presenting prenatally with skin edema and multisystem disease immediately after birth. Prenat Diagn. 2002;22:34‐37. 11810647 10.1002/pd.232

[12] Genetics Home Reference . Leigh syndrome. https://ghr.nlm.nih.gov/condition/leigh-syndrome. Accessed June 2016.

